# Leçons De Gynecologie Operatoire

**Published:** 1889-03

**Authors:** 


**Leçons de Gynecologie Operatoire.** Par VULLIET et LUTAUD, avec, 180 figures (448 pages). Paris: Bailliére et fils. 1889.

Seldom since we first perused Sims have we had such genuine pleasure in reading a gynecological work. Though in the shape of lectures, nothing is left out. Vulliet contributes fifteen and Lutaud eight lectures. The numerous wood-cuts greatly enhance the value of the book, actually representing almost half of it, although their execution is frequently very rough. Their main importance is the originality of the majority of the drawings. The language is lucid, the minutiæ not neglected, and thus the work well fit for the beginner. Several peculiarities, worthy of mention, are: The dilatation of the uterus up to such a degree that the cavity thereof assumes the form of a conus, with the apex at the fundus—"la grande dilatation;" the good results of uterine massage; the rules about treating sterility, and the good results of the actual cautery in cancer, minor points not included. The only novelty some may miss is the electrolytic proceedings of Apostoli.

A very beautiful way of reasoning brings Vulliet to the conclusion that in normal conditions the pelvic viscera, and especially the uterus, suffer only the most insignificant pressure from above. We have to refer in this respect to the original.

The book is certainly very worthy of translation into the English language. The *grande dilatation* being original with Vulliet, deserves special description. If the uterus is very narrow, he begins, after a thorough antiseptic cleansing of the vagina, with a tent or metallic dilator, or both. After the first expansion, he substitutes repeated tamponning of the uterus with iodoformized cotton plugs attached to threads. He uses iodoform, one to thirty ether, to dip the plugs in. After evaporation of the ether the plug, which is never above the size of a hazelnut, is used, and such a number of them packed into the uterus as the latter will hold. After forty-eight hours, the plugs are removed and new ones substituted, and this proceeding so often repeated until the funnel is obtained which can be inspected, by a speculum of his own, way into the fundus. At the time when Vulliet used stronger solutions, he saw a few cases of beginning iodoform-poisoning. The asepsis is thus maintained well for weeks; one difficulty may be met with—slipping out of the tampons—if the secretion of the uterus is profuse. All there is about it is replacing by new ones, which will stay, because the mucus is gone with the first ones. While engaged reading the book, we had a fit occasion to try this proceeding in a case of a broad-based intra-uterine papilloma, but abandoned it on account of the long time required, preferring the pain of more rapid, though less complete, dilatation to this painless method. We had to pay one penalty though, by the greater difficulty in the final operation.

